# A239 PATIENT OUTCOMES AFTER ENDOSCOPIC STENT INSERTION FOR MALIGNANT BILIARY OBSTRUCTION: A QUALITY ASSURANCE STUDY

**DOI:** 10.1093/jcag/gwac036.239

**Published:** 2023-03-07

**Authors:** T Krahn, S Veldhuyzen van Zanten, A J Montano-Loza, S Zepeda-Gomez, V Bain, R Sultanian, J -E Nilsson, S T Wasilenko, G Sandha

**Affiliations:** Division of Gastroenterology and Hepatology, University of Alberta, Edmonton, Canada

## Abstract

**Background:**

Management of pancreaticobiliary malignancy is complex and multi-disciplinary. Decompression of malignant biliary obstruction (MBO) is preferentially achieved with endoscopic retrograde cholangiopancreatography (ERCP) and biliary stent placement. This may improve the quality of life for patients with unresectable disease and improve outcomes in resectable/borderline-resectable disease.

**Purpose:**

To assess quality outcomes in patients undergoing biliary stenting for MBO.

**Method:**

This is a retrospective chart audit of patients referred to the University of Alberta Hospital (UAH) for suspected or confirmed MBO. The primary outcome was clinical success (reduction in bilirubin of >50% at 30 days). Secondary outcomes were technical success, type of stent used, need for re-intervention, adverse events (AEs), time from stent placement to surgery or cancer centre assessment, and survival.

**Result(s):**

Between January 2020 and June 2022, 222 patients (102 female, 46%) with a mean age of 70±1 years (range 34-93 years) underwent 290 ERCPs. The cause of MBO was pancreatic cancer in 130 (59%), cholangiocarcinoma in 32 (14%), ampullary cancer in 9 (4%), and others in 51 (23%).

Technical success for stent insertion on first ERCP was achieved in 180/222 patients (81%) with only brushings performed in 2. Of the 40 patients (18%) with unsuccessful ERCP, further success was achieved by repeat ERCP (8), percutaneous transhepatic drainage (PTC, 15), PTC with rendezvous ERCP (1), surgical decompression (3), and endoscopic ultrasound-guided biliary drainage (1). No biliary drainage was performed in 12 patients. Overall, ERCP with stent insertion was technically successful in 188/222 patients (85%).

A total of 233 biliary stents were inserted (38 plastic, 195 metal). Clinical success was achieved in 20/38 patients (53%) with plastic stents and 151/181 patients (83%) with metal stents (Χ^2^ 17.4 p<0.05).

Adverse events were encountered in 33 patients (11%) with stent migration occurring in 8 (3%), cholangitis in 7 (2%), post-ERCP pancreatitis in 7 (2%), post-sphincterotomy bleeding in 4 (1%), and death in 2 (1%). Re-intervention was required in 36 patients (20%) after initial ERCP with successful stent placement. The re-intervention rate was significantly higher with plastic stents (14/27, 52%) than with metal stents (22/153, 14%) after initial ERCP (Χ^2^ 20.1 p<0.001).

The overall survival was a mean of 249±16 days (5-967) for patients with plastic stents and 248± 16 days (1-959) for those with metal stents. Survival was significantly worse in patients with unresectable disease vs resectable/borderline-resectable disease (Log rank 38.89, p<0.001), Figure 1.

**Image:**

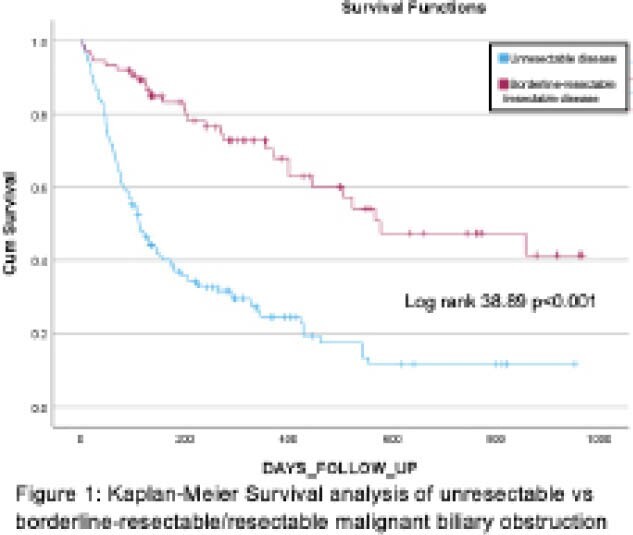

**Conclusion(s):**

In MBO, metal stents appear to provide significantly better biliary drainage, with less need for re-intervention, but do not appear to be associated with any survival benefit over plastic stents. We hope that this quality assurance project will help in the development of a regional management pathway for optimizing the care of patients presenting with MBO.

**Please acknowledge all funding agencies by checking the applicable boxes below:**

None

**Disclosure of Interest:**

None Declared

